# Exercise and Cleanliness

**Published:** 1890-01

**Authors:** Emma Howard Wight


### EXERCISE AND CLEANLINESS.

BY EMMA HOWARD WIGHT.

One of the greatest factors to preserve health is open air exercise, and another, essential to beauty, is cleanliness, which is indeed next to Godliness. Some one will, perhaps, exclaim in regard to the latter point, "Oh, that cannot be. Look at the Gypsies, and some uncivilized races, who pay no attention whatever to cleanliness, and yet among them you will find splendid specimens of beauty." Very true, but is it not a beauty to which "distance lends enchantment?" It is quite a charming picture to stand off and look at a Gypsy encampment, the splendid forms of the men, the rich, dark beauty of the women, the bright, picturesque costumes, the dark, dimpled, half-clad, little children tumbling about on the grass. But approach nearer and if you are fairly fastidious, the charm vanishes. You lose sight of the fact that the men are splendidly formed, the women darkly beautiful, the costumes picturesque, and the babies dark-skinned Cupids, when you see the uncleanliness which pervades all. The homeliest milk-maid, going for her cattle, in her spotless calico dress, with her fresh, clean skin, would be a far lovelier sight, and one to which you would turn with relief and pleasure.

As cleanliness is a promoter of beauty, it would be well to speak of the subject of bathing. I know to advocate frequent bathing is to go against the principles of not a few physicians, also against the practice of our grandmothers, who would never have dreamed of breaking their fixed rule, of a weekly Saturday night bath, and, perhaps, suggestions of one during the week. But, though we have been frequently told that the world has degenerated since their young days, and that *they* never did this thing or that thing, that girls do nowadays, (which is very true, no doubt,) nevertheless, in these latter days of more frequent bathing, I think we have, in this respect, improved on them. In the first place, the body is constantly throwing off poisonous matter, especially during sleep, which accumulates on the cutisvera, or outer skin, and without frequent bathing, the system re-absorbs this poisonous matter which certainly cannot be conducive to health.

Physicians maintain that frequent bathing is bad for persons with weak constitutions, but can it improve the poor health of these persons for the system to re-absorb the poisonous mattter thrown off by the body, probably more poisonous because of their weak or diseased condition? The frequent sponging of the body in a solution of alcohol and water is most beneficial. A quick cold sponge bath and an energetic rubbing of the body with a flesh brush or coarse towel are most invigorating to a weak constitution, if taken daily and systematically, thus bringing all the muscles into play and sending the blood tingling through the veins. A warm bath taken before retiring, cleansing the skin of the poisonous matter thrown off, as well as of the dust and dirt accumulated during the day is soothing and resting to the tired nerves and induces sleep. The Turkish bath has become quite the fashion. It is a luxury in itself, and most beneficial to the complexion. Some physicians prescribe it for certain kinds of diseases, but others are eloquent in their denunciations of it. A celebrated dermatologist of England says that the reason that some physicians oppose it is that the Turkish bath will both cure and prevent disease by simple and natural means. A cold plunge in the morning, on arising, is also of great benefit to the complexion.

Exercise in the open air is not only conducive to good health, but is absolutely necessary to it. Why is it that the children of the poor are so much rosier and healthier than the children of the rich? It is because they spend the greater part of their lives in the open air. For the same reason the country girl has brighter eyes, fresher complexion, and is healthier and stronger than the city girl. The farmer's son, too, has more strength and vigor that the city bred young man. If people would believe it, exercise in the open air is the best of doctors. He who avails himself of it will seldom have to resort to drugs. It will also effect a cure where drugs have failed. If one of my sex is suffering from nervousness let her abandon medicines, which do her no good, and take a long, brisk walk daily, and she will find that her nervousness will rapidly disappear, or, if deeply rooted, be much modified. If one suffers from insomnia, a walk in the afternoon, or if possible, just before retiring, will do more towards wooing sleep than any other course.

It is a great mistake to keep delicate, sickly children closely confined to the house. Send them out into the open air, do not wait until the weather is warm, but wrap them up well and send them out into the cold. Cold, dry weather will never hurt anybody properly clad. Let your children run and play in the fresh air, and they will soon be healthy, fat, and rosy. Why is it that boys are so healthy, so tough and hard to hurt? Simply because they spend so much of their time racing out of doors. It is highly necessary that school children should have plenty of out door exercise. Nothing is more injudicious than to crowd the brain and stint the body. Again, exercise is essentially necessary for the brainworker. It makes the brain clearer and more active, doing away with nervousness, headache, weariness, or any of the bad effects which follow a straining of the mind, and the worker returns to his task refreshed in mind and bcdy, ready and eager to take up that work which he had almost given up in despair, with new stimulus and reinvigorated ideas and strength. Exercise prolongs a woman's youth by keeping up her vitality, and retards the signs of the growing years by keeping her skin clear, fresh, and unwrinkled, and her eyes bright.

Of late years much attention has been paid to physical culture. There are gymnasiums in the schools, and home. The latest "fads" among women are gymnastics, lawn tennis, rowing, cycling, walking and horse back riding. It is fashionable to row, to use dumbbells, ride a bicycle and walk long distances. Not to make a pretense of doing these things, but to do them thoroughly and in earnest. The society girl no longer desires to be considered frail and delicate, but healthy and strong. She does not mind getting her hands sunburned and rough, or disguising her figure in a loose blouse. And when she looks in her mirror and sees the bright eyes and lovely flush exercise has brought to her, she never gives a thought to her sunburnt hands, which time and a little care will remedy, and she would be a very silly girl if she did. It is true her figure does not show to advantage in the loose blouse, nor her foot in the low-heeled lawn-tennis shoe, but they enable her to do thoroughly and comfortably whatever of the latest "fads" she may engage in. Winter has now come, and rowing, cycling, and lawn tennis will, to a great extent, retire to the background, so that out-of-door exercise will be confined to horseback riding and walking. The former is very delightful, but the latter far more beneficial; indeed no form of exercise can take its place, because, in walking, the whole body is exercised, and every muscle brought into play.

It is considered part of the society girl's education to be taught to dance. It is more to the point to teach her first to walk well and gracefully. Go along the streets of a city and the majority of girls you meet are awkward walkers, ungraceful in carriage. Often in a street car one will see pretty, attractive looking girls, but as soon as they arise the charm vanishes. They are awkward walkers, or, perhaps, round shouldered. Nothing detracts more from a girl's appearance than this latter defect. The face may be beautiful, but round shoulders are a deformity for which there is no excuse. Any one can remedy this defect by following this simple rule: preserve an upright position when walking, make an effort to grow a little taller, straighten the back, throw out the chest and hold the head high. After a little practice this position will become habitual.

